# 免疫检查点抑制剂在驱动基因阳性晚期非小细胞肺癌中的应用进展

**DOI:** 10.3779/j.issn.1009-3419.2021.101.04

**Published:** 2021-03-20

**Authors:** 同梅 张, 宝兰 李

**Affiliations:** 101149 北京，首都医科大学附属北京胸科医院综合科，北京市结核病胸部肿瘤研究所 Beijing Chest Hospital, Capital Medical University, Beijing Tuberculosis and Thoracic Tumor Research Institute, Beijing 101149, China

**Keywords:** 肺肿瘤, 驱动基因, 免疫检查点抑制剂, 预测, Lung neoplasms, Oncogene, Immune Checkpoint Inhibitors, Predictor

## Abstract

随着肿瘤精准医学的发展，驱动基因阳性非小细胞肺癌（non-small cell lung cancer, NSCLC）患者接受靶向治疗极大地改善了生存和预后，但不管是哪代靶向药物均不可避免的会经历耐药，患者会面临无靶可用的局面。免疫检查点抑制剂因其特有的长拖尾效应，能给部分晚期NSCLC患者带来长生存。越来越多的研究显示免疫治疗同样可为部分驱动基因阳性NSCLC患者带来获益，但免疫治疗介入的时机、治疗方案的选择以及预测生物标记物等问题仍不十分明确，值得进一步探讨。

## Abstract (en)

With the development of precise medicine, targeted therapy has greatly improved the survival and prognosis of patients in advanced non-small cell lung cancer (NSCLC) with oncogenic drivers. However, no matter which kinds of targeted therapy are inevitable to develop therapeutic resistance, treatment options upon exhaustion of targeted therapies are limited. Immune checkpoint inhibitors (ICIs) can bring long-term survival to some patients with advanced NSCLC because of its unique long tailing effect. More and more studies have shown that ICIs can also benefit NSCLC patients with oncogenic drivers. However, the timing of ICIs intervention, the therapeutic regimen and the predictive biomarkers are actually debated, underscoring the need to explore the potential interest of ICIs in these populations.

## 1 背景及现状

随着分子生物学的迅速发展，非小细胞肺癌（non-small cell lung cancer, NSCLC）的诊断和治疗发生了很大的变化。第一个表皮生长因子受体（epidermal growth factor receptor, EGFR）酪氨酸激酶抑制剂（tyrosine kinase inhibitor, TKI）吉非替尼开启了NSCLC患者EGFR-TKI治疗的新时代。免疫检查点抑制剂（immune checkpoint inhibitors, ICIs）已广泛应用于多种肿瘤的治疗^[1]^。针对程序性死亡因子1（programmed cell death 1, PD-1）及程序性死亡因子配体1（programmed cell death ligand 1, PD-L1）为靶点的ICIs能将晚期NSCLC患者的5年生存率提高至15%及以上^[2-4]^，ICIs正在全面改变NSCLC患者的治疗格局。

回顾众多ICIs的临床研究，多数研究均将驱动基因阳性尤其是EGFR突变和间变性淋巴激酶（anaplastic lymphoma kinase fusion gene, *ALK*）融合基因的患者排除在外。回顾性的研究亦显示*EGFR*、*ALK*等驱动基因改变的患者从单药ICIs的治疗中获益有限^[5]^，个案报道或临床实践中又能观察到部分驱动基因阳性的患者能从单药ICIs的治疗中获益^[6-9]^。临床实践中，对于驱动基因阳性的NSCLC患者来说，ICIs治疗的时机、优选方案、预测标志物等仍不十分清楚，值得进一步探讨。

## 2 NSCLC常见驱动基因改变的免疫原性

与肿瘤发生发展相关的基因水平的分子改变，称为肿瘤驱动基因。随着第二代测序技术（next generation sequencing, NGS）的广泛应用，发现了越来越多的功能性驱动基因，其中*EGFR*、鼠类肉瘤病毒癌基因（Kirsten rat sarcoma viral oncogene, *KRAS*）、间变淋巴瘤激酶融合基因（anaplastic lymphoma kinase fusion gene, *ALK*）最为常见。另外ROS原癌基因1（ROS proto-oncogene 1, *ROS1*）、间质上皮转换因子基因（mesenchymal-epithelial transition factor, *MET*）、RET原癌基因（RET proto-oncogene, *RET*）、人表皮生长因子受体2（man eoidermal growth factor receptor-2, *HER2*）和鼠肉瘤病毒v-Raf同源致癌基因（v-Raf murine sarcoma viral oncogene homolog, *BRAF*）等靶点药物也进入临床，针对这些驱动基因开展的靶向治疗极大地改善了此类患者的预后^[10]^。

肿瘤驱动基因的改变在NSCLC的疾病进展中起重要的作用，癌基因的改变可以通过减少肿瘤抗原的表达和减少肿瘤床中的T细胞浸润来促进免疫抑制的肿瘤微环境形成。Akbay等^[11]^研究显示，癌基因*EGFR*途径的激活可上调PD-L1的表达和其他免疫抑制因子而重塑免疫微环境，而在激活*EGFR*突变的NSCLC细胞系中EGFR抑制剂降低了PD-L1的表达。在另一项临床前研究中，EGFR激活上调PD-L1，导致肿瘤细胞中T细胞凋亡，但并未观察到EGFR-TKI和抗PD-1抗体协同杀伤肿瘤细胞的作用^[12]^。由此可推测EGFR致癌途径激活中的免疫逃逸主要是通过EGFR激活上调PD-L1介导的。

## 3 驱动基因阳性NSCLC患者后线接受单药ICIs治疗的回顾性研究

回顾性研究显示单药免疫治疗在驱动基因尤其是EGFR/ALK阳性的晚期NSCLC患者中的疗效有限。IMMUNOTARGET研究^[5]^是较早在欧美开展的一项多中心回顾性研究，最终纳入551例驱动基因阳性（包含*KRAS*、*EGFR*、*BRAF*、*ALK*、*ROS1*、*RET*、*HER2*）的NSCLC患者，其中KRAS阳性患者占49%，EGFR阳性患者占23%。94.6%的患者接受TKI或化疗进展后接受单药ICIs治疗，客观缓解率（objective response rate, ORR）分别26%（KRAS）、24%（BRAF）、17%（ROS1）、16%（MET）、12%（EGFR）、7%（HER2）、6%（RET）、0%（ALK），总体偏低；全组中位无疾病进展生存期（median progression-free survival, mPFS）为2.8个月，中位生存期（median overall survival, mOS）为13.3个月。比较而言，*KRAS*突变患者治疗获益最大，mPFS为3.2个月，ALK阳性患者未观察到疗效，ORR为0%，mPFS为2.5个月。随后亦有针对EGFR、ALK、ROS1阳性的NSCLC患者接受ICIs的回顾性研究、真实世界研究和meta分析^[13-15]^，均一致地显示了单药ICIs治疗驱动基因阳性的NSCLC患者获益有限。

*MET*基因是NSCLC的驱动基因之一，主要包括基因突变、融合和扩增。Sabari等^[16]^对24例*MET*外显子跳跃突变的患者接受免疫治疗，ORR为17%（95%CI: 6%-36%），mPFS为1.9个月（95%CI：1.7个月-2.7个月）。Yoshimura等^[17]^报道了6例患者使用ICIs单药治疗，3例部分缓解（partial remission, PR）患者中有2例MET过表达。*BRAF*突变患者接受ICIs治疗的疗效尚不明确，Dudnik等^[18]^回顾性分析了39例*BRAF*突变的NSCLC患者，22例患者接受了ICIs治疗，*BRAF* V600E组对比非V600E组，ORR为25% *vs* 33%，mPFS为3.7个月*vs* 4.1个月。同时发现*BRAF*突变的NSCLC患者PD-L1高表达、TMB中低水平、微卫星稳定（microsatellite stable, MSS），提示*BRAF*突变NSCLC患者能从ICIs治疗中获益。

Guisier等^[19]^对21项研究中心107例携带*BRAF*、*HER2*、*RET*和*MET*基因改变的二线及以上的NSCLC接受ICIs治疗的数据进行分析，结果显示全组患者的中位缓解时间、PFS和OS分别为15.4个月、4.6个月和16.2个月。该研究提示在真实世界中，ICIs治疗BRAF、HER2、RET和MET阳性的NSCLC患者的临床获益与非选择NSCLC患者接近。Ichihara等^[20]^对58例接受ICIs治疗的EGFR阳性的NSCLC临床特征进行分析，结果显示前线EGFR-TKI治疗缓解时间 > 6个月的患者接受ICIs的疗效差于缓解时间 < 6个月的患者（PFS：5.3个月*vs* 12.1个月，*P*=0.002, 5），提示对于*EGFR*突变的NSCLC患者前线对EGFR-TKI的反应时间能预测ICIs的疗效。

回顾性研究和*meta*分析似乎向人们展示了单药ICIs在驱动基因阳性NSCLC患者后线治疗的疗效差强人意，但无论如何，仍有部分EGFR/ALK阳性患者能从ICIs治疗中获益。研究^[21-23]^显示*KRAS*、*KRAS*合并*TP53*等基因改变更能从免疫治疗中获益。综合现有文献数据，驱动基因阳性的NSCLC患者在靶向治疗进展后可以选择ICIs治疗，单药ICIs治疗在部分患者中能看到生存获益，尚缺乏预测疗效的生物标志物。

总之，回顾性研究中多数患者接受单药ICIs的治疗，90%以上患者接受的是PD-1抑制剂。尚不能回答不同种类的ICIs以及ICIs联合不同治疗方案是否能提高疗效。在回顾性研究中尚缺乏另一个ICIs细胞毒T淋巴细胞蛋白4（cytotoxic T-lymphocyte-associated protein 4, CTLA-4）抑制剂在驱动基因阳性晚期NSCLC中的相关报道。

## 4 单药ICIs治疗驱动基因阳性NSCLC的前瞻性临床研究

回顾性临床研究因受限于样本量、患者入组偏倚和研究设计等问题，证据级别受到限制。ICIs治疗驱动基因阳性的晚期NSCLC的疗效和安全性在设计严谨的前瞻性临床研究能更好的明确其临床意义。一项针对未使用过EGFR-TKI且PD-L≥1%的NSCLC接受单药Pembrolizumab的Ⅱ期临床研究因疗效有限而提前终止^[24]^。ATLANTIC研究^[25]^是一项评估Durvalumab治疗三线及以上晚期NSCLC的Ⅱ期研究，根据基因突变状态和PD-L1表达水平分为3个队列，在EGFR/ALK阳性PD-L1 > 25%的NSCLC患者队列中，mOS达13.3个月，EGFR阳性组mOS长达16.1个月，治疗相关的不良反应9.5%，患者耐受性总体良好。此研究为EGFR/ALK阳性晚期NSCLC靶向治疗耐药后的治疗提供新的选择。值得注意的是此研究与回顾性研究的不同点在于，研究用药为PD-L1抑制剂，入组患者PD-L1表达 > 25%。因此，驱动基因阳性NSCLC患者应用ICIs治疗PD-L1是否为预测因子？PD-1抑制剂与PD-L1抑制剂是否不同？尚需更多前瞻性临床研究来回答。

## 5 ICIs联合治疗驱动基因阳性NSCLC的临床研究

前期研究或回顾性研究似乎提示单药ICIs治疗驱动基因阳性NSCLC患者获益有限，此类患者真的无ICIs治疗的机会吗？越来越多的证据表明，ICIs联合治疗能给驱动基因阳性NSCLC患者带来新的治疗选择，而联合治疗药物的种类、联合治疗的时机到底如何？

### 5.1 PD-1/PD-L1抑制剂联合TKI治疗

有关TKI联合ICIs的临床研究主要集中在EGFR/ALK阳性NSCLC患者中，而联合ICIs的种类主要集中在PD-1/PD-L1抑制剂中，旨在探索联合治疗在EGFR-TKI耐药后和初诊患者中的安全性和疗效^[26]^。一项吉非替尼联合Durvalumab治疗未接受过EGFR-TKI治疗的EGFR阳性患者的Ib期研究，队列1接受吉非替尼联合Durvalumab同步治疗，队列2（10例患者）在吉非替尼治疗4周后开始联合治疗，研究显示3级/4级副反应主要为谷丙转氨酶（alanine aminotransferase, ALT）和天冬氨酸转氨酶（aspartate aminotransferase, AST）升高，仅1例出现间质性肺炎，队列1和2的ORR分别为77.8%和80%^[27, 28]^。另一个Ib期临床研究^[29]^评估了28例未接受过EGFR-TKI的EGFR阳性NSCLC患者一线接受Atezolizumab联合厄洛替尼治疗，ORR为75%，未出现剂量限制性毒性（dose limiting toxicities, DLTs），无间质性肺炎发生，3级/4级TRAE发生率为39%（ALT升高为7%，皮疹为7%，发热为7%）。在一项多中心Ⅰ期临床研究（NCT02088112）中，入组一线使用吉非替尼联合PD-L1抑制剂Durvalumab治疗初诊EGFR阳性的晚期NSCLC患者，结果显示联合治疗并未改善PFS，但毒副反应增加，35%的患者因肝毒性终止治疗^[30]^。CheckMate370研究^[31]^的E队列纳入了13例接受Nivolumab联合克唑替尼治疗的ALK阳性NSCLC患者，5例（38%）患者出现严重的肝毒性，其中2例死亡，试验因此终止。

随后亦有研究在RGFR-TKI耐药后的患者中探索TKI联合ICIs治疗的疗效和安全性。一项多队列临床研究^[32]^纳入21例EGFR阳性的NSCLC患者（20例厄洛替尼进展，1例初治）接受厄洛替尼联合Nivolumab治疗，ORR为19%，mPFS为29.4周，1年生存率为73%，24%者为3级毒性（主要为肝细胞溶解、腹泻和无力）。此研究还纳入20例*KRAS*突变患者，治疗反应率与*EGFR*或*KRAS*突变状态无关，*EGFR*和*KRAS*突变患者PFS和OS较野生型短。

CAURAL研究（NCT02454933）是一项多队列研究，显示在EGFR-TKI治疗失败的T790M阳性晚期NSCLC患者中对比奥西替尼联合Durvalumab和单药奥西替尼，因ORR改善有限（单药组*vs*联合组为80% *vs* 64%），最终因联合治疗导致的间质性肺炎的增加而提前终止^[33]^。随后其子队列TATTON研究（NCT02143466）评估奥西替尼联合Durvalumab治疗，23例EGFR-TKI失败的EGFR阳性患者入组A组（剂量递增），ORR在T790M阳性组和阴性组分别为67%和21%；11例未接受EGFR-TKI治疗患者入组B组（扩展队列）ORR为70%，两组因严重的间质性肺炎的发生率升高而被叫停（A组为38%，B组为64%）^[34]^。在一项Ib期临床研究^[35]^中评估了Atezolizumab联合Cobimetinib治疗初诊的实体瘤患者，其中包含了28例NSCLC患者，ORR为18%，提示联合治疗安全可控，疗效与*KRAS*/*BRAF*突变状态无关。

随后开展的TKI联合ICIs治疗的临床研究显示联合治疗并不能改善ORR，尤其在初诊患者中联合治疗因副反应而终止研究的也不少见。鉴于TKI联合ICIs治疗相关毒副反应的发生率较高，该联合治疗模式仍存在争议，尚需进一步探索，有关TKI联合ICIs的临床研究汇总，见表 1。

**1:** TKI联合ICIs治疗驱动基因阳性NSCLC临床研究汇总 Summary of clinical study of TKI combined with ICIs in drive gene-positive NSCLC

NCT No.	Biomarker	TKI	ICIs	Stage	Status
NCT02039674	EGFR	Erlotinib/Gefitinib	Pembrolizumab	Ⅰ/Ⅱ	Ongoing
NCT02088112	EGFR	Gefitinib	Durvalumab	Ⅰ	Ongoing
NCT1454102	EGFR	Erlotinib	Durvalumab	Ⅰ	Ongoing
NCT02143466	EGFR	Osimertinib	Durvalumab or Durvalumab+Tremelimumab	Ⅰ	Ongoing
NCT02364609	EGFR	Afatinib	Pembrolizumab	Ⅰ	Ongoing
NCT02013219	EGFR	Erlotinib	Atezolizumab	Ⅰ	Ongoing
NCT02574078	EGFR	Erlotinib	Nivolumab	Ⅰ/Ⅱ	Ongoing
NCT04141644	EGFR	Osimertinib	Ipilimumab	Ⅰb	Recruiting
NCT04538378	EGFR	Olaparib	Durvalumab	Ⅱ	Recruiting
NCT02349633	EGFR (Del19 or L858R±T790M)	PF-06747775, Palbociclib	Avelumab	Ⅱ	Terminated
NCT02630186	EGFR	Rociletinib	MPDL3280A	Ⅰb/Ⅱ	Terminated
NCT02898116	ALK	Ensartinib	Durvalumab	Ⅰ/Ⅱ	Terminated
NCT02511184	ALK	Crizotinib	Pembrolizumab	Ⅰ	Terminated
NCT02393625	ALK	Ceritinib	Nivolumab	Ⅰ	Active, not recruiting
NCT03299088	KRAS	Trametinib	Pembrolizumab	Ⅰ	Recruiting
NCT03004105	KRAS	Selumetinib	Durvalumab	Ⅱ	Withdrawn
NCT04613596	KRAS G12C	MRTX849	Pembrolizumab	Ⅱ	Recruiting
NCT03785249	KRAS G12C	MRTX849	Pembrolizumab	Ⅰ/Ⅱ	Recruiting
NCT04449874	KRAS G12C (Arm B)	GDC-6036	Atezolizumab	Ⅰ	Recruiting
TKI: tyrosine kinase inhibitor; ICIs: immune checkpoint inhibitors; NSCLC: non-small cell lung cancer; EGFR: epidermal growth factor receptor; NCT: National Clinical Trial.					

### 5.2 CTLA-4抑制剂联合TKI治疗

迄今少有CTLA-4抑制剂Ipilimumab联合TKI的临床研究报道。一项Ipilimumab联合厄洛替尼或克唑替尼治疗的Ⅰ期试验（NCT01998126）^[36]^，以评估联合治疗对EGFR或ALK阳性的晚期NSCLC患者的安全性。研究要求EGFR或ALK阳性的NSCLC患者服用稳定剂量的厄洛替尼或克唑替尼超过28 d才有可参加研究，入组患者需接受联合4个周期的Ipilimumab 3 mg/kg治疗，在EGFR队列中因腹泻毒副反应将Ipilimumab减量至1 mg/kg，最终因4例3级结肠炎，在入组11例患者后关闭研究。在ALK队列的3例患者中，出现1例垂体炎，1例2级肺炎。但经过长期随访后发现EGFR阳性队列的mPFS为27.8个月，mOS尚未达到，ALK阳性队列mPFS为24.1个月，mOS尚未达到。该研究显示厄洛替尼或克唑替尼联合Ipilimumab可引起毒性反应的增加，但PFS和OS数据较单药TKI令人关注。

### 5.3 双免疫联合治疗

在一项不同剂量Pembrolizumab联合Ipilimumab 1 mg/kg的临床研究^[37]^中纳入了二线及以上EGFR/ALK阳性的NSCLC患者，其中39例EGFR阳性患者和1例ALK阳性患者。在Pembrolizumab 2 mg/kg联合Ipilimumab 1 mg/kg队列中的10例EGFR阳性患者，1例获得客观缓解，ORR为10%，缺乏后续数据更新报道。由此可见，双免疫治疗此类患者尚缺乏数据支持，尚待进一步研究。

### 5.4 化疗±抗血管联合ICIs治疗

IMpower150研究^[38]^评估化疗联合贝伐珠单抗（标准治疗组）、化疗联合Atezolizumab（3药组）和化疗联合贝伐珠单抗联合Atezolizumab（4药组）在未接受过化疗的Ⅳ期非鳞NSCLC，包括既往TKI治疗失败或不耐受的EGFR/ALK阳性NSCLC中的有效性和安全性。在EGFR/ALK阳性的亚组中，4药组对比3药组的中位PFS为10.2个月*vs* 6.9个月，风险比（hazard ratio, HR）为0.60。这一研究结果提示，4药方案治疗TKI治疗失败的*EGFR*/*ALK*阳性的晚期非鳞NSCLC能给患者带来临床获益，中位OS达29.4个月^[39]^。此研究为免疫联合治疗驱动基因阳性NSCLC开启了新的治疗模式选择。亦有个案报道^[40]^显示EGFR-TKI耐药后含铂紫杉醇联合贝伐珠单抗及Atezolizumab的4药联合方案的显著的疗效。Qiu等^[41]^对真实世界接受PD-1抑制剂联合抗血管患者进行分析，亚组分析显示EGFR阳性组患者的PFS达5.4个月，虽然小于EGFR阴性患者的10.5个月，但较单药ICIs治疗有所改善，提示ICIs联合抗血管亦是此类患者的一种治疗选择。

在驱动基因阳性NSCLC中，靶向药物进展后有接受ICIs治疗的机会，多种ICIs联合不同治疗方案的注册临床研究正在开展中，详见表 2。

**2:** ICIs联合其他治疗驱动基因阳性NSCLC临床研究汇总 Summary of clinical study of ICIs combined with other therapy to drive gene-positive NSCLC

NCT No.	Biomarker	Treatment	Stage	Status
NCT04517526	*EGFR* mutations after failure of first line osimertinib	Durvalumab+Pemetrexed+platinum+bevacizumab+salvage SBRT	Ⅱ	Not yet recruiting
NCT04470674	*Kras* mutation positive and PD-L1 high	Durvalumab±Pemetrexed+carboplatin	Ⅱ	Not yet recruiting
NCT04426825	*EGFR* mutation positive Stage Ⅲb/Ⅳ non-squamous	Atezolizumab+Bevacizumab	Ⅱ	Recruiting
NCT04322591	PD-1 antibody in RET fusion positive	Chemotherapy±PD-1 inhibitors	-	Recruiting
NCT04245085	*EGFR*-mutant NSCLC	Arm A: Atezolizumab+Bevacizumab+Pemetrexed+Carboplatin Arm B: Atezolizumab+Bevacizumab +Paclitaxel	Ⅱ	Recruiting
NCT04147351	*EGFR*-mutant metastatic NSCLC patients after failure of *EGFR*-TKI	Atezolizumab+Bevacizumab	Ⅱ	Recruiting
NCT04120454	*EGFR* mutant NSCLC	Pembrolizumab+Ramucirumab	Ⅱ	Recruiting
NCT04099836	*EGFR* mutant NSCLC with progressive disease after receiving osimertinib	Atezolizumab+Bevacizumab	Ⅱ	Recruiting
NCT04042558	Non-squamous NSCLC with *EGFR* mutation, ALK rearrangement or ROS1 fusion progressing after targeted therapies	Carboplatin+Pemetrexed+Atezolizumab±Bevacizumab	Ⅱ	Recruiting
NCT03994393	Advanced *EGFR* mutant NSCLC	Tremelimumab+Durvalumab	Ⅱ	Recruiting
NCT03991403	*EGFR* mutation or ALK translocation NSCLC	Atezolizumab+Bevacizumab+Carboplatin+Paclitaxel vs Pemetrexed+Carboplatin /Cisplatin	Ⅲ	Recruiting
NCT03802240	*EGFR*-mutant +non-squamous NSCLC patients after *EGFR*-TKI failure	Sintilimab+Pemetrexed+Cisplatin±IBI305	Ⅲ	Recruiting
NCT03765775	NSCLC patients with first-generation *EGFR*-TKI resistance along with T790M negative	Sintilimab+Anlotinib	Ⅱ	Recruiting
NCT03647956	*EGFR*-mutant metastatic NSCLC patients after failure of *EGFR*-TKI	Atezolizumab+Bevacizumab+Carboplatin+Pemetrexed	Ⅱ	Recruiting
NCT03515837	TKI-resistant *EGFR*-mutated non-squamous NSCLC	Pemetrexed+Platinum±Pembrolizumab	Ⅲ	Active, not recruiting
NCT03256136	*EGFR*-mutant or *ALK*-rearranged NSCLC	Nivolumab+Carboplatin+pemetrexed vs Nivolumab+Ipilimumab	Ⅱ	Completed
NCT03091491	*EGFR*-mutant NSCLC	Nivolumab vs Ipilimumab+Nivolumab	Ⅱ	Unknown
NCT02864251	NSCLC with *EGFR* mutation failed 1Lor 2L *EGFR*-TKI	Nivolumab+chemotherapy vs Nivolumab+Ipilimumab vs Chemoterapy	Ⅲ	Active, not recruiting

## 6 展望

免疫治疗正在改变着肺癌的治疗格局，对于有相应靶点药物的驱动基因阳性的晚期NSCLC患者，在靶向治疗进展且无靶向药物可用时，单药ICIs治疗能为部分患者带来生存获益，不同驱动基因接受ICIs治疗疗效存在差异。而免疫联合化疗和/或抗血管治疗能让更多驱动基因阳性NSCLC患者获益，尚缺乏大型的前瞻性临床研究数据。在生物标志物的研究中，尚缺乏驱动基因阳性NSCLC接受ICIs疗效预测生物标志物。对于驱动基因阳性的NSCLC免疫治疗最佳的模式是在精准生物标志物的指导下的治疗，还需要深入开展基础和临床研究，为更多的驱动基因阳性NSCLC带来更多的生存获益。
